# Healthcare burden changes by restricted physical activities in lumbar spinal stenosis and spondylolisthesis: a retrospective large cohort study during the COVID-19 pandemic

**DOI:** 10.1186/s12891-024-07332-1

**Published:** 2024-05-24

**Authors:** Jun-Hoe Kim, Yebin Chegal, Suhyun Kim, Hangeul Park, Young Rak Kim, Sum Kim, Kwangsoo Kim, Chang-Hyun Lee, Chi Heon Kim, Chun Kee Chung

**Affiliations:** 1 Department of Neurosurgery, Seoul National University Hospital, Seoul, South Korea; 2 Department of Statistics, Korea University, Seoul, South Korea; 3 Transdisciplinary Department of Medicine & Advanced Technology, Seoul National University Hospital, Seoul, South Korea; 4 Department of Neurosurgery, Seoul National University College of Medicine, 101 Daehak-ro, Jongro-gu, Seoul, 03080 South Korea; 5 Department of Brain and Cognitive Sciences, Seoul National University, Seoul, 03080 Republic of Korea

**Keywords:** Lumbar spinal stenosis, Spondylolisthesis, COVID-19, Common data model

## Abstract

**Background:**

Lumbar spinal stenosis (LSS) and spondylolisthesis (SPL) are characterized as degenerative spinal pathologies and share considerable similarities. However, opinions vary on whether to recommend exercise or restrict it for these diseases. Few studies have objectively compared the effects of daily physical activity on LSS and SPL because it is impossible to restrict activities ethnically and practically. We investigated the effect of restricting physical activity due to social distancing (SoD) on LSS and SPL, focusing on the aspect of healthcare burden changes during the pandemic period.

**Methods:**

We included first-visit patients diagnosed exclusively with LSS and SPL in 2017 and followed them up for two years before and after the implementation of the SoD policy. As controls, patients who first visited in 2015 and were followed for four years without SoD were analyzed. The common data model was employed to analyze each patient’s diagnostic codes and treatments. Hospital visits and medical costs were analyzed by regression discontinuity in time to control for temporal effects on dependent variables.

**Results:**

Among 33,484 patients, 2,615 with LSS and 446 with SPL were included. A significant decrease in hospital visits was observed in the LSS (difference, -3.94 times/month·100 patients; *p* = 0.023) and SPL (difference, -3.44 times/month·100 patients; *p* = 0.026) groups after SoD. This decrease was not observed in the data from the control group. Concerning medical costs, the LSS group showed a statistically significant reduction in median copayment (difference, -$45/month·patient; *p* < 0.001) after SoD, whereas a significant change was not observed in the SPL group (difference, -$19/month·patient; *p* = 0.160).

**Conclusion:**

Restricted physical activity during the SoD period decreased the healthcare burden for patients with LSS or, conversely, it did not significantly affect patients with SPL. Under circumstances of physical inactivity, patients with LSS may underrate their symptoms, while maintaining an appropriate activity level may be beneficial for patients with SPL.

## Introduction

Degenerative lumbar spinal diseases, including lumbar spinal stenosis (LSS) and spondylolisthesis (SPL), are some of the most common, with an estimated global prevalence of 130 million individuals globally [1]. Although LSS and SPL reduce walking distance due to radicular leg pain and neurogenic claudication, the pathogenesis of the two diseases differs to some extent. LSS is a degenerative condition characterized by spinal canal narrowing, leading to leg symptoms [2–4]. SPL is characterized by the displacement of a vertebra in a forward or backward direction relative to the vertebra beneath it. Spinal instability is a major pathogenesis of SPL and may exacerbate back pain by weakening back muscles [5–8]. Although LSS and SPL share considerable similarities, opinions vary on whether to recommend exercise or restrict it for these diseases [3, 4, 9–17]. Concerning exercise, evidence of treatment effects for LSS and SPL is different. For SPL, several papers have reported that exercise effectively controls pain and improves function [15, 17]. Regarding LSS, a randomized controlled trial found that exercise did not show a competitive advantage in patients with LSS [18]. Few studies that objectively compare the effects of daily physical activity on LSS and SPL exist because it is impossible to restrict activities ethically and practically.

**Fig. 1:**
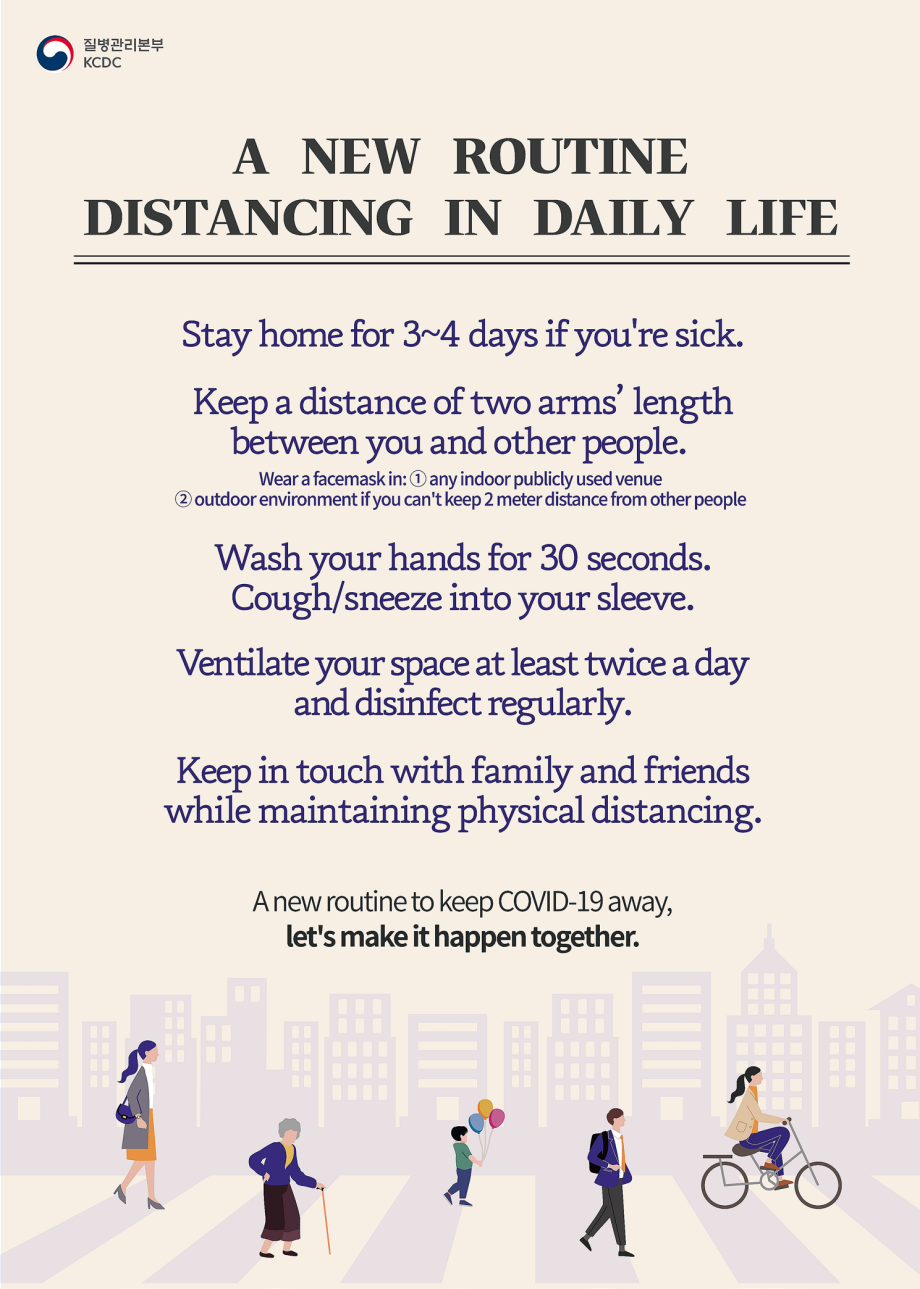
Social distancing guidelines in the republic of korea implemented in march 2020

Coronavirus disease 2019 (COVID-19), caused by the severe acute respiratory syndrome coronavirus 2 (SARS-CoV-2), emerged in December 2019 in Wuhan, China, and quickly became a global pandemic. In January 2020, the first imported case of COVID-19 was confirmed in the Republic of Korea [19]. From March 2020, the Korean government activated social distancing (SoD) quarantine policies to prevent the transmission of COVID-19. Many sports facilities, such as fitness clubs and swimming pools, closed for approximately 2 years (Fig. 1) [20]. The government forced people to “stay at home” to reduce person-to-person transmission and imposed strong restrictions on outdoor physical activity. This policy led to a substantial decrease in physical activity, although there might have been individual variations in adherence to this policy [21]. During the SoD period, the incidence of some diseases, such as obesity, increased, while others, such as trauma, decreased [22, 23].

The aim of this study is to investigate the effect of restricting physical activity due to SoD on LSS and SPL, focusing on the aspect of healthcare burden changes such as hospital visits and medical expenditures during the COVID-19 pandemic.

## Materials and methods

### Study design and data source

This large retrospective cohort study assessed changes in the number of hospital visits and medical expenditures among all patients diagnosed with LSS and SPL who visited our hospital before and after the SoD policy. We employed big data techniques utilizing the common data model (CDM) adopted by the Observational Health Data Sciences and Informatics (OHDSI) network. The diagnostic codes, treatments, medications, and procedures for each patient were standardized within the CDM as concept identification numbers. This enabled the acquisition of information such as the time of diagnosis and administration of drugs or treatments. Demographic information, including year and month of birth and sex, was also obtained from the CDM.

This study was conducted in accordance with the Declaration of Helsinki. The protocol was reviewed and approved by the Institutional Research Board (E-2303-052-1411). The Institutional Research Board approved the exemption of patient informed consent owing to the retrospective nature of this study.

### Patient selection

To compare medical behavior in the 2 years before and after March 2020, we included first-visit patients diagnosed with SPL and LSS without SPL in 2017. Only patients with LSS or SPL who visited the departments of neurosurgery, orthopedic surgery, rehabilitation medicine, anesthesiology (pain clinic), and radiology (pain intervention) were included in the analysis to count only hospital visits for the treatment of LSS or SPL. The exclusion criteria were (1) under the age of 40; (2) patients with diagnostic codes (M60-99) indicating soft tissue diseases, osteopathies, chondropathies, and connective tissue diseases; (3) patients with diagnostic codes (Q00-99) indicating congenital deformities; (4) patients with diagnostic codes C or D corresponding to tumorous conditions; and (5) ≤ 2 visits during the follow-up period. The cohort was followed up for 2 years (2018–2019) without a SoD policy and then for the next 2 years (2020–2021) with a SoD policy. To delineate the usual treatment pattern without SoD, we defined the control groups as those diagnosed with LSS or SPL for the first time in 2015 with 4 years of follow-up.

### Outcome variables

A visit was defined as an event when the LSS and SPL diagnostic codes were registered. The calculation of medical costs involved extracting the concept identification numbers corresponding to a variety of procedures, including epidural steroid injections, decompressive laminectomy, and spinal fusion, as well as those for disease-related medication and radiologic examinations (lumbar X-rays, computed tomography, and magnetic resonance imaging). The frequency of each procedure was determined through this data extraction. Subsequently, the costs for each procedure were calculated by applying the standard unit costs for the year 2022, as determined by the National Health Insurance Service of the Republic of Korea, to their respective frequencies. From these calculated medical costs, we determined the median medical cost per visit for each month and utilized it as a variable in our analysis. Additionally, demographic and diagnostic data, including age, sex, COVID-19 diagnosis, and diabetes mellitus diagnosis, were also extracted.

#### Statistical analysis

This study used the standardized Observational Medical Outcomes Partnership (OMOP) CDM (version 5.3). The effect of time of SoD policies was estimated using a regression discontinuity (RD) analysis. RD is a statistical technique allowing researchers to identify causal effects by controlling for observable and unobservable factors [24]. The method involves using a running variable, also known as a rating variable or assembly variable, which is a continuous variable affecting treatment or outcome. In this study, as the results were affected by time, an analysis was conducted by setting time as the running variable. This specific application of RD is called regression discontinuity in time (RDiT) [25]. RD can eliminate selection bias, as the assignment of units to exposure of SoD is based on a cutoff, where any unit above the cutoff receives the exposure and units below do not. In this study, the implementation of SoD measures served as the cutoff for the running variable and the washout period of the policy was defined as 3 months. The aim was to identify significant change as called as “jump” in the number of hospital visits and monthly medical costs for patients following the implementation of the cutoff. The RD design includes assumptions that must be met for the analysis to be valid:

1. Continuity assumption: It is important to note that all other observable variables are assumed to be continuous at the cutoff point.
2. No sorting across the cutoff: It is also assumed that units cannot perfectly sort themselves across the cutoff; for instance, farmers cannot sell land to become eligible.

There is a difference between sharp and fuzzy regression discontinuity regarding the assignment rule; the assignment rule of sharp regression discontinuities is deterministic, and that of fuzzy regression discontinuities is probabilistic [26]. In this study, SoD measures were implemented nationally and globally; therefore, the sharp regression discontinuity design was adopted.

Let ${Y}_{i}^{1}$, ${Y}_{i}^{0}$, and ${X}_{i}$ be the potential outcomes and running variable, respectively, for subject $i$. ${D}_{i}$ is the intervention. Then, the observed outcome equation is written as:

$${Y}_{i}= {Y}_{i}^{0}+\left({Y}_{i}^{1}- {Y}_{i}^{0}\right){D}_{i}$$

As ${Y}_{i}^{0}$ is allowed to depend on ${X}_{i}$ through a linear model, the equation is given as:

$${Y}_{i}= {\alpha }+ {\beta }{X}_{i}+ \delta {D}_{i}+ {\epsilon }_{i}$$

where ${\alpha }$ and ${\beta }$ are regression coefficients, including the interception and ${\epsilon }_{i}$ is the random error for subject $i$.

$$\delta = {Y}_{i}^{1}- {Y}_{i}^{0}$$

$$\delta = \underset{{X}_{i}\to {X}_{0}}{\text{lim}}E\left[{Y}_{i}^{1}\right| {X}_{i}={X}_{0}]- \underset{{X}_{0}\leftarrow {X}_{i}}{\text{lim}}E\left[{Y}_{i}^{0}\right| {X}_{i}={X}_{0}]$$

$$= \underset{{X}_{i}\to {X}_{0}}{\text{lim}}E\left[{Y}_{i}\right| {X}_{i}={X}_{0}]- \underset{{X}_{0}\leftarrow {X}_{i}}{\text{lim}}E\left[{Y}_{i}\right| {X}_{i}={X}_{0}]$$

δ is the treatment effect parameter, which indicates a discontinuity in the conditional expectation and is considered the average causal effect of the treatment.

Suppose that ${D}_{i}=I({X}_{i}\ge {c}_{0})$ and ${c}_{0}$ is the cutoff. Then, the observed outcome equations of sharp RD using linear and nonlinear models are

Linear estimation

$$Y_i=\alpha+\beta\left(X_i-c_0\right)+\delta D_i+\epsilon_i$$

Nonlinear estimation

$${Y}_{i}=\alpha + {\beta }_{1}{(x}_{i}-{c}_{0})+{\beta }_{2}{\left({x}_{i}-{c}_{0}\right)}^{2}+ \cdots + {\beta }_{p}{\left({x}_{i}-{c}_{0}\right)}^{p}+\delta {D}_{i}+{\eta }_{i}$$

where ${\beta }_{1}, \dots , {\beta }_{p}$ are the corresponding coefficients and ${\eta }_{i}$ is the random error for subject $i$. A standardized mean difference was used to compare demographic characteristics, such as age and sex, for homogeneity.

## Results

**Fig. 2:**
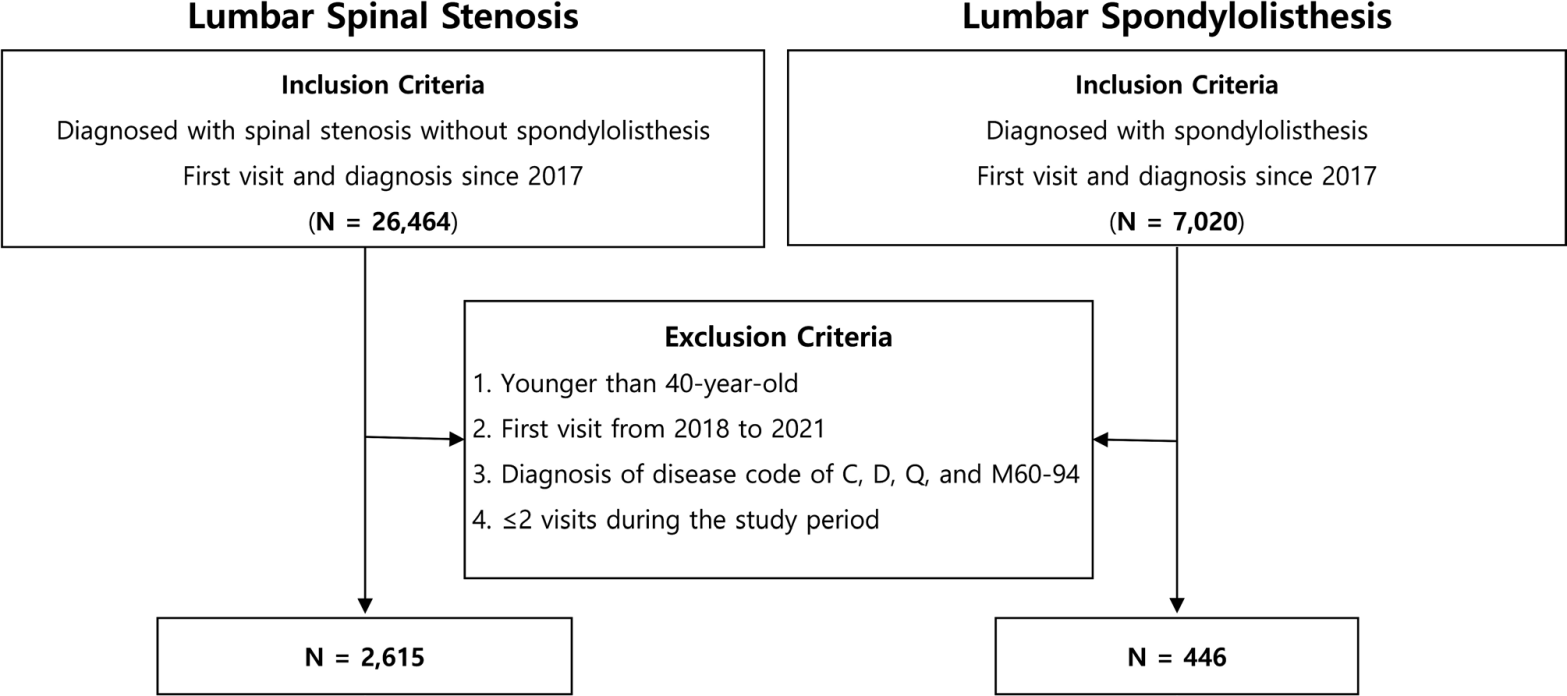
Flow diagram of included patients

In 2017, the number of patients who visited our hospital for the first time was 26,464 in the LSS group and 7,020 in the SPL group, with 19 doctors providing medical care. After applying the exclusion criteria, 2,615 patients with LSS and 446 patients with SPL were included in this study (Fig. 2). The control group comprised patients who first visited our hospital in 2015, and it had 2,049 patients with LSS and 332 patients with SPL. The baseline characteristics between the two study periods in each disease group were similar, except for age and sex in the SPL group (Table 1).

**Table 1:** Baseline characteristics of patients

Variables	First Visit Year	LSS	SPL	P-value
Number of patients	2015	2,049	332	
	2017	2,615	446	
Number of Female	2015	1260 (61.5%)	260 (78.3%))	< 0.001^*^
	2017	1580 (60.4%)	340 (76.2%)	< 0.001^*^
Mean age	2015	70.0 ± 9.6	66.8 ± 9.1	< 0.001^*^
	2017	71.3 ± 9.8	69.4 ± 9.6	< 0.001^*^
Diagnosis of COVID-19	2015	22 (1.1%)	6 (1.8%)	0.381
	2017	33 (1.3%)	6 (1.3%)	1
Diagnosis of DM	2015	129 (6.3%)	24 (7.2%)	0.601
	2017	184 (7.0%)	41(9.2%)	0.13
Diagnosis of tumor	2015	231 (11.3%)	45 (13.6%)	0.266
	2017	366 (14.0%)	66(14.8%)	0.707

Values are presented as mean ± standard deviation or number (%). Statistical analysis between the two groups by chi-square test or two sample t-test (Abbreviations: LSS, lumbar spinal stenosis; SPL, spondylolisthesis; DM, diabetes mellitus)

**Fig. 3:**
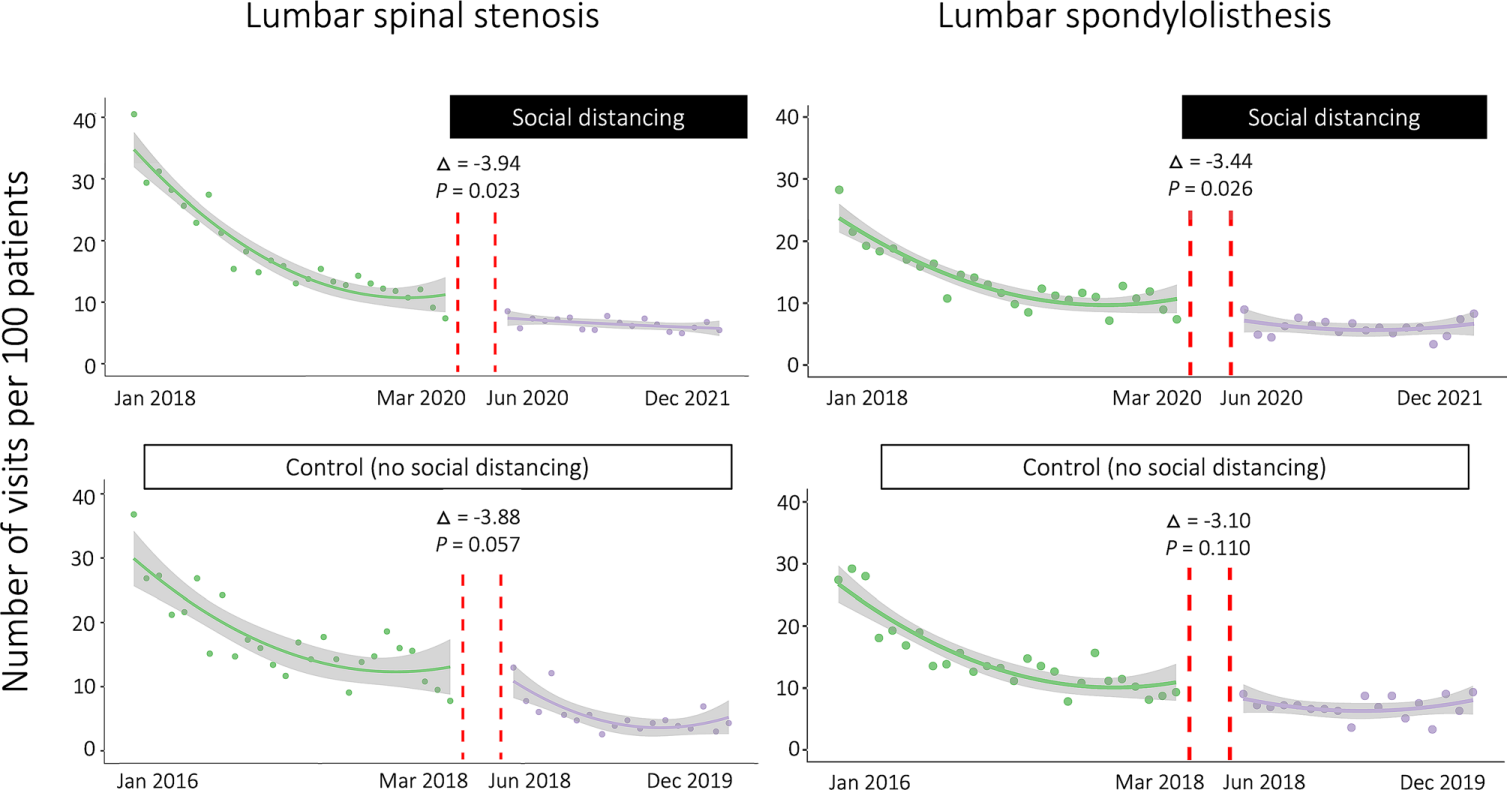
Scatter plots with regression lines depicting the number of hospital visits. The number of visits for spinal stenosis and spondylolisthesis significantly decreased after social distancing (upper). In comparing the natural course of these diseases, no significant change was observed in the number of visits 2 years prior to the social distancing period

After enrolling patients who visited the hospital for the first time in 2017, we observed that the number of visits for LSS and SPL was nearly 40 per 100 patients and 30 per 100 patients in the first month of the follow-up period (January 2018). This gradually decreased to 10 per 100 patients by the end of 2019, as shown in Fig. 3. According to the regression discontinuity in time (RDiT) analysis, significant decreases in the number of hospital visits were observed in the LSS (difference, -3.94; standard error, 1.67; *p* = 0.023) and SPL (difference, -3.44; standard error, 1.49; *p* = 0.026) groups after the implementation of the SoD policy. In the control patients enrolled in 2015 who were unaffected by SoD, the number of hospital visits gradually decreased in both the LSS and SPL groups. However, the control patients did not display a significant discontinuity in the number of hospital visits in either the LSS (*p* = 0.057) or SPL (*p* = 0.110) groups.

**Fig. 4:**
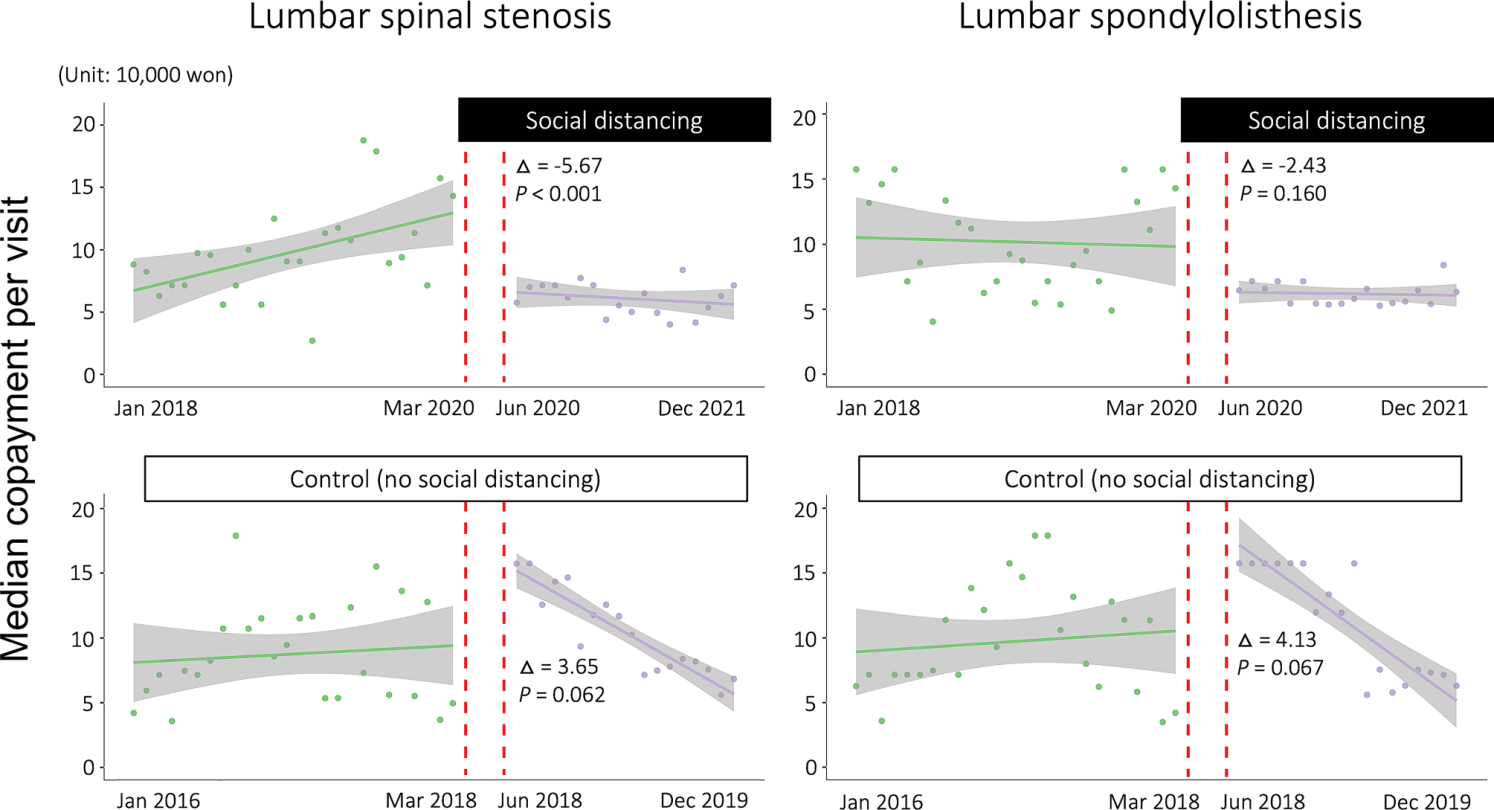
Scatter plots with regression lines depicting the median copayment per visit. After social distancing from March 2020, the medical costs for spinal stenosis decreased significantly (*p* < 0.001), and that for spondylolisthesis did not show substantial change (*p* = 0.160). In the 2 years before social distancing, no difference was seen in the change in medical costs for both diseases

Concerning medical costs, both the LSS and SPL groups in the control group showed that the median copayment per visit did not change significantly for the 2-year follow-up period and then decreased gradually. In the middle of the follow-up period, there was no significant discontinuity of LSS (*p* = 0.062) or SPL (*p* = 0.067) treatment between 2015 and 2019. Conversely, for the patients who were enrolled in 2017, the median copayment per visit in the LSS group significantly decreased by approximately 56,500 KRW (USD 45) after the SoD policy (Fig. 4) (standard error, 1.47; *p* < 0.001). However, the SPL group did not show a significant difference (difference: 24,300 KRW [USD 19]; standard error, 1.70; *p* = 0.160) in the median copayment per visit after the SoD policy.

## Discussion

This study evaluated the effect of restricting physical activity on two degenerative lumbar diseases. We controlled for confounding factors, including major comorbidities, age, and less than 2 visits. To determine the causal effect of SoD while controlling for other unobservable factors, we employed an RDiT analysis. After physical activity was restricted by the SoD policy during the pandemic period, the number of visits in the LSS and SPL groups significantly decreased compared with the period without SoD. The median copayment per visit during SoD was significantly reduced in the LSS group but not in the SPL group.

Routine treatment for LSS and SPL without SoD in our hospital was observed in the control group in the study period from 2016 to 2019. After the first visit for LSS or SPL in 2015, the visit number showed a logarithmic decline for 2 years from 40 to 10% of patients and then a slight decrease for 2 years (Fig. 3). For the first 2 years, 90% of the enrolled patients might have ended treatment at our hospital. The remaining 10% of patients visited our hospital continuously, indicating that these patients complained of chronic pain and periodically received medication and injections. Regarding medical costs, there was a gradual increase during the initial two years, followed by a decrease. Higher medical expenses seemed to be attributed to surgical and pain interventions in the early stages of treatment (Fig. 4).

Although both variables were influenced by time, the washout period of 3 months did not make a significant statistical difference in either disease. After the SoD policy was implemented in March 2020, there was a significant decrease in the number of hospital visits among patients with LSS and SPL compared with the period without the SoD policy. This reduction occurred despite no government-imposed restrictions on clinic or hospital visits during the SoD period. This observed trend of decreased medical service usage, particularly in spine surgery, is supported by findings from several studies [27–30]. However, the median copayment per visit showed a difference between the groups. While the median copayment per visit significantly decreased in the LSS group, there was no change in the SPL group. If the decreased intention to utilize healthcare services because of social aspects is similar in both groups, other factors might account for this difference. Considering that the costs for operative treatment are higher than for non-operative treatment in lumbar degenerative disease [31] and that patients with more severe symptoms are more likely to undergo surgery [32], it is possible that there were differences in symptom progression between LSS and SPL patients after the initiation of SoD. Brembilla et al. [33], who also reported a significant decrease in emergency room visits for LSS, suggest a possible explanation. During the SoD period with reduced physical activity, patients may underrate their symptoms, which implies that the perceived symptoms in patients with LSS might have decreased. Meanwhile, given that limited physical activity can exacerbate musculoskeletal pain [34–36], it is plausible that reduced physical activity significantly impacted the worsening of SPL symptoms.

Differences in the pain mechanism in LSS and SPL may explain this discrepancy. Two theories explain the neurogenic claudication pain generation in LSS. One is the ischemic theory, which postulates decreased arterial flow in the nerve root, and the other is the venous stasis theory, which postulates inadequate oxygenation and accumulation of metabolites due to venous stasis [2, 37]. Neurogenic claudication pain may not occur if patients do not walk for a long enough time, a situation that might apply to those with LSS during the SoD period. Conversely, in SPL, it is believed that pain is caused not only by neurogenic claudication but also by instability, resulting in strain on the erector spinae muscles. These factors can cause symptoms even when not walking for a long time by simply changing posture or moving indoors [5, 7]. Furthermore, during the SoD period, weakening of the erector spinae muscles due to limited physical activity might have led to the worsening of SPL symptoms. This observation aligns with previous reports showing that exercise therapy often demonstrates greater effectiveness in SPL [15, 16, 18].

Limited physical activity increases various metabolic risks and negatively impacts degenerative diseases [38, 39]. In this era of post-SoD, we do not suggest that reducing physical activity is recommended for improving symptoms in patients with LSS. However, before considering high-cost and high-risk treatments like surgery, it is crucial to evaluate changes in the activity levels of patients with LSS to avoid overrating their symptoms [33]. Additionally, for patients with SPL, it is advisable to adopt a more proactive approach in educating them about home exercise programs to maintain an adequate level of physical activity during a potential situation where physical activity is constrained [40–42].

Our study had several limitations. First, this big data study attempted to control for other variables, excluding SoD and LSS/SPL, but it might not have been entirely perfect. To control for other confounding factors, we excluded the patients with major comorbidities such as tumors, congenital disease, and connective tissue diseases. The reason is that patients with these conditions might have visited spine clinics more readily while they were at the hospital because of these diseases. With regard to the severity of LSS or SPL, we could not acquire the data from the CDM. This study included all patients who met the inclusion criteria among approximately 1.5 million annual outpatient visits, which might have minimized sampling bias. However, it can be a limitation of this study. In addition, it is hard to distinguish the purpose of analgesics for the management of cancer-related pain or rheumatic diseases. Despite our best efforts, we cannot say that all variables other than SoD have been controlled. However, this study may hold significance in evaluating the impact of physical activity on LSS or SPL, considering the ethical impossibility of enforcing restrictions on outdoor activities to determine the effects of exercise.

Secondly, interpreting changes in hospital visits and medical expenses may not accurately reflect the patient’s intention to utilize healthcare services or the severity of their symptoms. The possibility exists that enrolled patients visited another hospital. However, we anticipate that the underestimation owing to the follow-up loss of patients will be low. Our hospital did not reduce clinic schedules, and the government did not restrict movement for medical treatment during the pandemic. In addition, our hospital requires a referral for outpatient visits, and patients seeking care elsewhere are rare. The price increase in medication, outpatient visits, or relevant expenses due to inflation must be considered during the COVID-19 period. However, South Korea operates a social insurance system, and the government strictly controls medical costs. During the study period, the unit price increased 2.0 – 2.3% per year. Considering the slight increase in medical costs, it might have a minor effect on increasing medical expenditures. Moreover, patients are responsible for only 10 – 20% of total medical expenses, which results in a relatively low possibility of treatment abandonment due to the medical expense burden. The enrolled patients might have been infected with COVID-19, potentially influencing the study results. However, only 5 in the LSS group and 1 in the SPL group were diagnosed with COVID-19 prior to June 2020, when we analyzed discontinuity. Even when including patients infected after this period, they only constitute about 1% of each group. Therefore, it appears that the direct impact of COVID-19 can be considered minimal.

Thirdly, although a quantifiable decrease in the population’s activity levels was reported [21], specific figures on how much the study participants reduced their daily physical activity could not be ascertained. Thus, additional studies using large-scale, nationwide data are necessary to yield more reliable results.

## Conclusion

Restricted physical activity during the SoD period decreased the healthcare burden for patients with LSS or, conversely, it did not significantly affect patients with SPL. Under circumstances of physical inactivity, patients with LSS may underrate their symptoms, while maintaining an appropriate activity level may be beneficial for patients with SPL.

## Data Availability

The dataset analyzed during the current study are available from the corresponding author on reasonable request.
